# Canada lynx use of burned areas: Conservation implications of changing fire regimes

**DOI:** 10.1002/ece3.2824

**Published:** 2017-03-12

**Authors:** Carmen M. Vanbianchi, Melanie A. Murphy, Karen E. Hodges

**Affiliations:** ^1^ Department of BiologyUniversity of British Columbia OkanaganKelownaBCCanada; ^2^ Department of Ecosystem Science and ManagementUniversity of WyomingLaramieWYUSA

**Keywords:** Canada lynx, fire regime, habitat use, *Lynx canadensis*, North Cascade Mountains, predators, Random Forest models, Washington, wildfire

## Abstract

A fundamental problem in ecology is forecasting how species will react to major disturbances. As the climate warms, large, frequent, and severe fires are restructuring forested landscapes at large spatial scales, with unknown impacts on imperilled predators. We use the United States federally Threatened Canada lynx as a case study to examine how predators navigate recent large burns, with particular focus on habitat features and the spatial configuration (e.g., distance to edge) that enabled lynx use of these transformed landscapes. We coupled GPS location data of lynx in Washington in an area with several recent large fires and a number of GIS layers of habitat data to develop models of lynx habitat selection in recent burns. Random Forest habitat models showed lynx‐selected islands of forest skipped by large fires, residual vegetation, and areas where some trees survived to use newly burned areas. Lynx used burned areas as early as 1 year postfire, which is much earlier than the 2–4 decades postfire previously thought for this predator. These findings are encouraging for predator persistence in the face of fires, but increasingly severe fires or management that reduces postfire residual trees or slow regeneration will likely jeopardize lynx and other predators. Fire management should change to ensure heterogeneity is retained within the footprint of large fires to enable viable predator populations as fire regimes worsen with climate change.

## 1 Introduction

Climate change is inducing hotter, drier, and longer summers in North America. Consequently, hotter, larger, and more severe wildfires are burning (Balshi et al., 2009; Fauria & Johnson, 2007; Littell et al., 2010; Westerling, Hidalgo, Cayan, & Swetnem, 2006), and in 2015, the United States saw a record‐setting 4.1 million ha consumed (National Interagency Fire Center 2016). Fire suppression efforts also increased in 2014 and 2015; over $3.5 billion USD were spent on firefighting efforts (National Interagency Fire Center 2016). Boreal forests account for more than one‐third of global forest covering much of the circumpolar north, making an increase in the boreal fire regime significant not only for the economy (National Interagency Fire Center 2016) but for ecosystem services such as carbon storage (Brassard & Chen, 2006; Goldammer & Furyaev, 1996) and for wildlife habitat (Appenzeller, 2015).

Boreal forests are characterized by dramatic and frequent disturbances that create a continually shifting mosaic of successional stages across the landscape (Agee, 2000; Perera & Buse, 2014), and the most important boreal and sub‐boreal forest disturbance is wildfire (Agee, 2000). Wildfires burn millions of hectares per year in the boreal forest, often over large areas and at intensities that initiate stand replacement (Perera & Buse, 2014). These dramatic fires drive the boreal landscape's heterogeneity of forest age structure and species assemblages.

**Figure 1:**
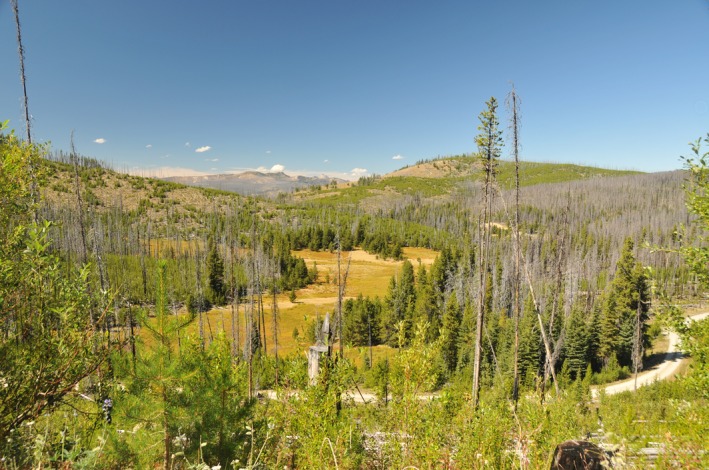
A postfire mosaic within the Tripod Burn in northcentral Washington, USA. The fire burned in 2006; this picture was taken in August 2016 (the radio‐collared lynx were on air from 2007 to 2013). Within the burn scar, there are wet meadows, dry meadows, fire skips where trees were not burned, dead trees, and areas with scattered to dense patches of young trees regrowing after the fire. Lynx are more likely to use areas with denser cover, whether the cover is derived from residual unburned material or areas with dense regeneration of trees or shrubs. Photograph copyright Karen E. Hodges

Boreal fires create heterogeneity both at the landscape level and within a single burn perimeter as fire behavior varies greatly according to weather, microclimate, fuels, and topography (Cansler & McKenzie, 2014; Perera & Buse, 2014) (Figure 1). As a result, some areas burn at a high intensity, consuming forest canopies and leaving only burnt snags behind, while other areas burn at a lower intensity such that the understory burns but many trees survive (Brassard & Chen, 2006; Perera & Buse, 2014). Fire skips, areas within a burn perimeter that do not burn at all, leave the original forest structure and species composition intact (Perera & Buse, 2014). Consequently, the composition of the residual vegetation and structural features such as live trees, snags, and downed logs fluctuates across a burn.

In turn, forest regeneration patterns vary, influenced by the presence or absence of residual vegetative reproductive structures such as coniferous seeds released from serotinous cones, underground suckers, or wind‐blown seeds from fire skips and burn edges (Brassard & Chen, 2006; Perera & Buse, 2014). Residual snags and logs also affect regrowth as they provide substrate, shade, and physical protection for young seedlings (Brassard & Chen, 2006). Finally, site‐specific variations in soils, climate, and topography also affect regeneration patterns and, combined with varying residual vegetation compositions, result in a heterogeneous landscape within a single fire perimeter (Bonnet, Schoettle, & Shepperd, 2005; Brand, 1991; Crotteau, Varner, & Ritchie, 2013; Franklin & Dyrness, 1973; Irvine, Hibbs, & Shatford, 2009; Perera & Buse, 2014; Turner, Romme, Gardner, & Hargrove, 1997).

With the onset of climate change, more frequent, larger, and more severe fires will increase the amount of forest in an open stand‐initiation stage (Balshi et al., 2009; Fauria & Johnson, 2007; Littell et al., 2010; Soja et al., 2007; Westerling et al., 2006) and change the composition and spatial patterns of residual vegetation, potentially homogenizing the landscape within a fire perimeter (Cansler & McKenzie, 2014). Warmer and drier summers could also change forest regeneration patterns following a fire by limiting the establishment and growth of plant species dependent on moist conditions (Littell et al., 2010).

A change in fire regime and regeneration patterns will likely affect the wildlife of boreal forests. Historically, as succession progresses, plant communities change in composition and structure, and animal communities shift in response to the changing habitat (Fisher & Wilkinson, 2005; Fox, 1983). For example, the snowshoe hare (*Lepus americanus*) is an important boreal prey species whose presence can be predicted based on a forest stand's developmental stage. Hares depend on high stem density forests to provide browse and cover, a feature primarily found in young stands and in old‐growth forests where canopy gaps promote a multilayered structure (Hodges, 2000a,b; Hodson, Fortin, & Belanger, 2011). Unfortunately, although responses of animals to fire are documented for some small mammals and birds, substantial information gaps exist regarding responses of larger prey species and carnivores to fire (Fisher & Wilkinson, 2005). This lack of information hinders both current conservation and management of boreal forest carnivores and the ability to adapt conservation strategies as fire regimes shift under climate change.

One such carnivore is the Canada lynx (*Lynx canadensis*), an iconic boreal forest species that depends on the snowshoe hare for prey and is thus closely linked to forest structure. Studies of lynx in Alaska, Canada, and to a lesser extent in the sub‐boreal regions of the contiguous US document general trends in lynx response to fire, but lack detailed information that could be used to improve lynx management and conservation (Koehler, 1990; Paragi, Johnson, Katnik, & Magoun, 1997; Staples, 1995). These studies describe lynx as selecting against recent burns in the open stage where shrubs and trees have not grown tall enough to provide cover and browse for snowshoe hares, especially during the winter when snow covers low understory structure, but have not probed in detail what habitat features lynx use when they are within a recent burn scar (Hodson et al., 2011; von Kienast, 2003; Koehler et al., 2008; Maletzke, Koehler, Wielgus, Aubry, & Evans, 2008). Recent, more detailed studies in sub‐boreal forests of the western US document high hare densities in regenerating stands with high sapling densities within 0‐2 decades postfire (Cheng, Hodges, & Mills, 2015; Hodges, Mills, & Murphy, 2009), raising the question of whether lynx also use burns more quickly after fire than previously detected with limited datasets.

As forest regeneration progresses, burns in an early‐stand development stage (2–4 decades postfire) are often composed of dense regenerating deciduous shrubs and conifer trees that provide quality snowshoe hare habitat and thus quality lynx habitat (Hodges, 2000b; Mowat & Slough, 2003; Paragi et al., 1997; Stephenson, 1984). Stands regenerating postfire that move into a late‐stand development stage, where a closed canopy inhibits understory growth and self‐thinning eliminates branches in the understory, do not provide good snowshoe hare and lynx habitat (Hodson et al., 2011; Koehler, 1990; Paragi et al., 1997). Forests in this late‐stand development stage may not provide understory conditions preferred by snowshoe hares and lynx until a disturbance resets forest succession by returning the area to the early‐stand development stage or until the forest matures into old growth so that canopy gaps form, encouraging shrub growth, and tree boughs provide understory cover (Hodson et al., 2011; Maletzke et al., 2008; Squires, Decesare, Kolbe, & Ruggiero, 2010). However, beyond these general descriptions of lynx response to fire, little detail is known about how lynx respond to different burn severities, to the heterogeneity of regeneration in a burned area, or to the spatial configuration of a burned area.

Here, we use Canada lynx as a case study for examining whether and how predators use recently burned areas. In addition to intrinsic interest and legal requirements for protecting this species, lynx typify forest predators because they use a range of habitats and are highly mobile with records of dispersing lynx moving up to 1,100 km (Mowat, Poole, & O'Donoghue, 2000). Canada lynx in the contiguous US occur at the southern edge of lynx range in low‐density populations and have been federally listed as Threatened since 2000 (USFWS 2000), but a Recovery Plan is still lacking.

Within Washington State, the North Cascade Mountains are designated as critical lynx habitat (USFWS 2014) and support one of the few remaining lynx populations in the contiguous US and the only resident breeding population in Washington (Stinson, 2001). According to a 2008 population model of Washington lynx habitat by Koehler et al. (2008), the state provided habitat for an estimated 87 lynx. Washington lynx use home ranges that average 88 km^2^ (Vanbianchi, 2015) and select sub‐boreal forest types on moderate slopes at elevations between 1,200 and 2,000 m (Koehler, 1990; Koehler et al., 2008; McKelvey, Ortega, Koehler, Aubry, & Brittell, 2000). Specifically, lynx in the North Cascades select old‐growth multilayer Engelmann spruce (*Picea engelmannii*)–subalpine fir (*Abies lasiocarpa*) forest (the climax sere of the *Abies lasiocarpa* Zone; Franklin & Dyrness, 1973), where canopy openings encourage dense understory growth and low‐reaching boughs create additional horizontal cover and forage for snowshoe hares (Hodges, 2000b; Koehler et al., 2008; Lewis, Hodges, Koehler, & Mills, 2011). Lynx also select young lodgepole pine (*Pinus contorta*) forest (often present as an early‐seral stage of the *Abies lasiocarpa* Zone; Franklin & Dyrness, 1973), where high stem densities support snowshoe hares (Koehler, 1990; McKelvey et al., 2000).

**Figure 2:**
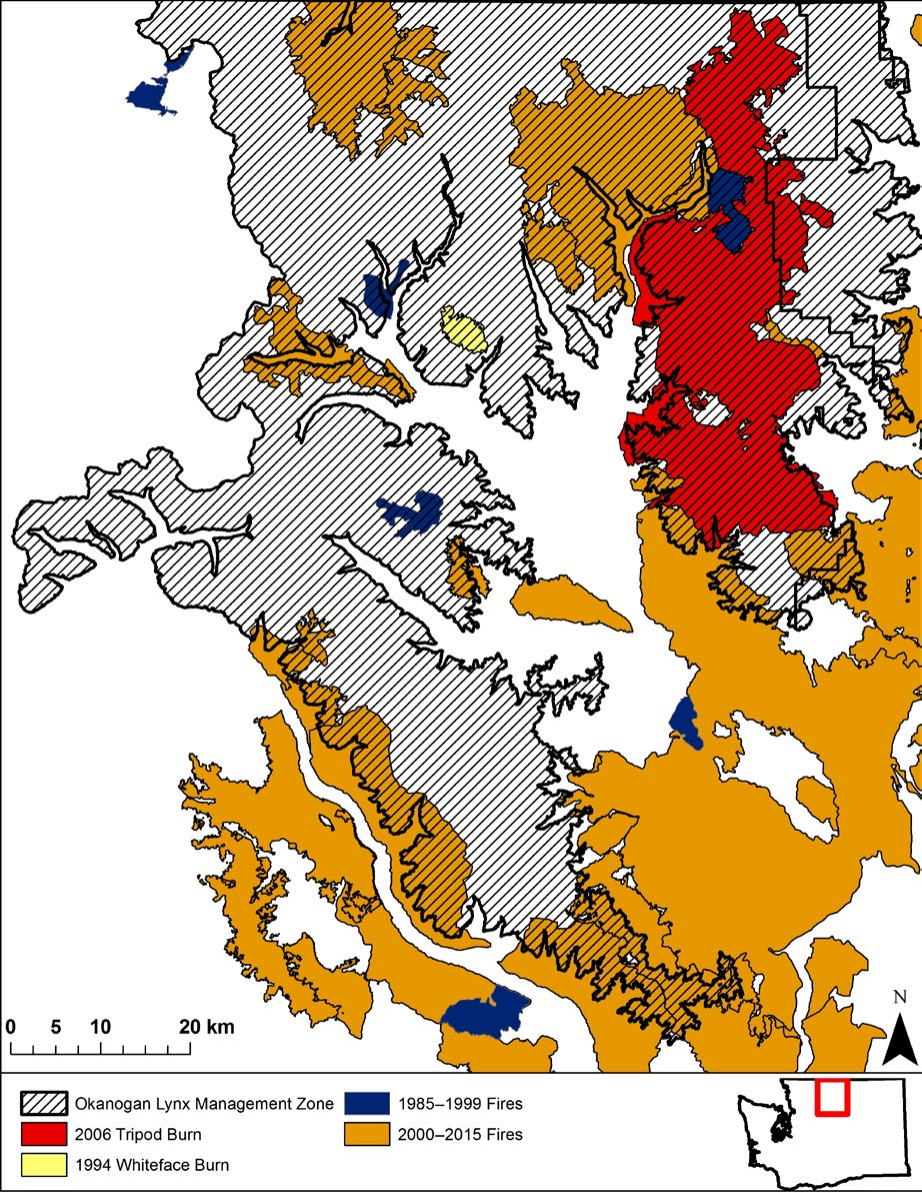
Large fires in northcentral Washington, Pacific Northwest USA, over the last 30 years. The Okanogan Lynx Management Zone is the only area in the state that retains a population of lynx. During the 1980s and 1990s, fires >1000 ha were considered large, but fires in the 2000s have been substantially larger. The top edge of the map is the Canada–Washington border

The North Cascades region has experienced a dramatic increase in wildfires over the last 30 years (National Interagency Fire Center 2016). In 1994, two fires of 1,554 ha and 3,686 ha were large relative to previous decades. Then, in 2003 and 2006, one fire burned 8,620 ha, and three fires burned >20,000 ha each (Figure 2). These fires have raised serious concerns about whether lynx populations will remain viable within the state; the state has uplisted lynx from Threatened to Endangered (Interagency Lynx Biology Team 2013; Lewis, 2016).

We examine (1) lynx use of burned areas 1–6 years and 17–19 years postfire in Washington and (2) what habitat features lynx selected within burned sites. We present results from 14 radio‐collared lynx (monitored 2007–2013) from the eastern slope of the North Cascades. In 2006, the large Tripod fire burned most of the prime lynx habitat in the state (Koehler et al., 2008; Stinson, 2001). We examine how lynx used the Tripod Burn and the 1994 Whiteface Burn. Because lynx behavior postfire is so poorly known, we used Random Forest models to determine which habitats lynx selected in this landscape, as this approach enables detection of unexpected patterns. We used lynx locations and spatial data (forest cover, topographic setting, climate, and burn history) as potential driving variables (Vanbianchi, 2015).

## 2 Methods

### 2.1 Study area

Our two study areas, the Whiteface and Tripod Burn study areas, are on the Eastern slope of the North Cascade Mountains in Washington and fall within the Okanogan‐Wenatchee Lynx Management Zone designated by the Washington State Lynx Recovery Plan (Stinson, 2001). The Whiteface Burn covers 1,554 ha in the Okanogan‐Wenatchee National Forest, Washington. Approximately 15 km east of the Whiteface Burn study area, the 70,644 ha Tripod Burn occurs within both the Loomis State Forest and the Okanogan‐Wenatchee National Forest (Figure 2). To match data from 14 radio‐collared lynx, we examined only the eastern portion of the Tripod Burn, a 46,800 ha area that includes the 1994 Thunder Mountain Burn (3,686 ha).

Cold, snowy winters and mild summers characterize the study areas, with average monthly temperatures in nearby Mazama, Washington (elevation: 664 m), ranging between −10°C and 23°C, with an average annual snowfall of 305 cm (Western Regional Climate Center, http://www.wrcc.dri.edu/, accessed June 20, 2014). Forest types range from sub‐boreal forests in high‐elevation areas and cool, mid‐elevation pockets and aspects, to low‐elevation dry forests (Lillybridge, Kovalchik, Williams, & Smith, 1995). The sub‐boreal forest consists of Engelmann spruce–subalpine fir forest or lodgepole pine forests. On warmer mid‐elevation sites, “mixed forests” transition from sub‐boreal types into a drier forest dominated by Douglas fir (*Pseudotsuga menziesii*), while lower elevations are dominated by “dry forests” of Douglas fir–ponderosa pine (*Pinus ponderosa*).

The Whiteface Burn ranges from 1,280 m elevation at its southern end to 2,222 m at the northern end, with an average of 1,650 m and 80% of its area above 1,500 m. Dry, mixed, and deciduous forest types cover 55% of the forested areas within the burn, largely at lower elevations. Sub‐boreal forest types exist at higher elevations and comprise 45% of the regenerating and residual forest. In the Whiteface Burn, 82% of the fire burned at a high severity (>50% canopy cover loss), while 10% burned at low severity (<50% canopy cover loss) and 8% of the area within the burn perimeter did not burn (Vanbianchi, 2015; Vanbianchi, Gaines, Murphy, Pither, & Hodges, unpublished data).

The Tripod Burn study area is higher than the Whiteface Burn, ranging from 855 m to 2,390 m with 93% of its area above 1,500 m. In contrast to the Whiteface Burn, the Tripod Burn study area has a large sub‐boreal forest component with 88% of the regenerating and residual forest type in this category. The Tripod Burn study area also has more lodgepole pine forest, which comprises 35% of the forest within the regenerating and residual forest category. In the Tripod Burn, 63% of the area burned at high severity and 8% burned at low severity. The Tripod fire nearly surrounded but did not reburn the 1994 Thunder Mountain Burn (3,686 ha), so 8% of the Tripod Burn study area is classified as an old (1985‐1997) burn. Fire skips in the Tripod Burn study area make up 21% of the burn and include a 1,850‐ha island of forest that has not burned since the 1970 Forks Fire (Vanbianchi, 2015; Vanbianchi et al., unpublished data).

### 2.2 Lynx data

Lynx data were provided to us courtesy of the Washington Department of Fish and Wildlife. Lynx were trapped and fitted with global positioning system (GPS) telemetry collars in the Okanogan ‐Wenatchee National Forest and the Loomis State Forest from January 2007 to April 2012. Trapping took place during the winter using box traps (Kolbe, Squires, & Parker, 2003) as a collaboration among the Washington Department of Fish and Wildlife, Washington Department of Natural Resources, U.S. Forest Service, U.S. Bureau of Land Management, and the U.S. Fish and Wildlife Service (ethics clearances and all necessary permitting were handled by these agencies). The collars were programmed to record GPS locations every 4 hr for 1 year, except for one collar programmed to record GPS locations every six hours. We used data from 14 adult lynx (three females and 11 males). Lynx were on air for varying durations, and the average fix rate was 72%; we also omitted data from dispersing or wandering lynx that left the study area. The average straight‐line distance travelled by a lynx in the four‐hour period between GPS fix attempts was 766 m (Vanbianchi, 2015; Vanbianchi et al., unpublished data).

### 2.3 Study area delineation

We used a raster dataset depicting wildfires in ArcGIS 10.1 (ESRI 2012) to define the perimeter of the Whiteface Burn. To outline the Tripod Burn study area, we used the raster dataset to define the eastern fire perimeter. All of the lynx with home ranges near the Tripod Burn resided on the eastern edge of the burn. Because the Tripod Burn extends further west than any of the nearby lynx ventured, we limited the western boundary by connecting sequential lynx locations with a straight line and then buffering the lines by the average step length (766 m) between GPS fixes. The outermost edge of the buffered lines was used to delineate the western extent of the Tripod Burn study area. To examine lynx habitat use in the Whiteface and Tripod Burn study areas, we used ArcGIS 10.1 (ESRI 2012) to generate random available locations within each study area equal to the number of used locations in each study area (Barbet‐Massin, Jiguet, Albert, & Thuiller, 2012).

### 2.4 Habitat variables

We used GIS layers to represent the landscape characteristics that are important to lynx habitat use (Table S1). We used ArcGIS 10.1 (ESRI 2012) to derive continuous representations of each predictor variable using 30 m^2^ pixels projected into the 1983 North American Datum Albers coordinate system. To explore habitat selection at different scales, we examined variables within 3 × 3 and 27 × 27 pixel windows (90 and 810 m width, respectively). Previous lynx research demonstrates lynx choose habitats both at fine scales and at larger patch or higher scales (Koehler et al., 2008; Maletzke et al., 2008). The 3 × 3 window is the smallest window we could use with our statistical approach and thus models the fine‐scale habitat selection. We then wanted this fine‐scale choice to nest within our large‐scale window; we chose a 27 × 27 window as more appropriate than a 9 × 9 window because previous research documents these animals are highly mobile and have large home ranges.

We categorized land cover into five forest types and three nonforest types (Table S1). Forest types were lodgepole pine and spruce‐fir, that is, sub‐boreal types known to be selected by lynx in the North Cascades (Koehler et al., 2008; Maletzke et al., 2008; McKelvey et al., 2000), “dry forest” (dominated by Douglas fir or ponderosa pine), “mixed forest” (transitional between sub‐boreal and dry types), and deciduous. Nonforested types were grassy meadows, shrubby meadows, and barren areas such as rock outcrops or ice fields. The land cover data categorized 23% of the Whiteface Burn simply as “disturbed,” based on residual trees providing <10% cover. To assign “disturbed” areas to one of our eight cover types, we used the ArcGIS 10.1 tool, Nibble, to assign “disturbed” pixels a land cover type that was based on the cover types of the surrounding pixels.

In high‐severity burned areas, only blackened tree trunks remain, while a low‐severity burn consumes understory cover but trees survive. To capture the effect of burn severity, we included variables depicting fire age and severity. Old burns burned in 1994 and included the Whiteface Burn and a burn within the Tripod fire scar that did not reburn. The new burn was the 2006 Tripod fire. Low severity was classified based on canopy cover loss of 1‐50%, while higher severity had >51% loss. We ended up with four categories of burn: old, high severity, old, low severity, new, high severity, and new, low severity (Table S1).

We examined how the spatial arrangement of burn pattern may influence lynx habitat selection by including a patch metric depicting the distance from each pixel within the burn to the nearest edge. We also modeled slope and the distance to the nearest draw, as both variables have evidence suggesting they affect lynx movement (Koehler et al., 2008; Maletzke et al., 2008; Stinson, 2001).

Lynx may select burned areas with a cool, moist climate where forest recovery can occur faster (Buskirk et al., 2000). Thus, we included the Compound Topographic Index as a measure of wetness based on the amount of upstream contributing area and slope (Gessler, Moore, McKenzie, & Ryan, 1995; Moore, Gessler, Nielsen, & Petersen, 1993), a Heat Load Index variable depicting temperature based on aspect and slope (McCune & Keon, 2002), and a variable for the average precipitation accumulated during the growing season. Finally, we had no understory cover GIS layer available for the study areas, so we used forest canopy cover as a proxy for the structure of a forest (Table S1).

### 2.5 Model development

We developed Random Forest (Breiman, 2001) habitat models for the Whiteface Burn and Tripod Burn by using *randomForest* (Liaw & Wiener, 2002) and *rfUtilities* (Evans & Murphy, 2014) packages in R software (Version 3.1.2, R Core team 2014). While Resource Selection Functions may be the predominant methodology for predicting and describing habitat use, a relatively new machine‐learning algorithm, Random Forest (Breiman, 2001), has recently been applied to habitat analysis studies (Mochizuki & Murakami, 2013; Wilsey, Lawler, & Cimprich, 2012). Random Forest has several advantages over Resource Selection Functions; it is nonparametric, and it accounts for interactions among variables and across scales, and the complex nonlinear relationships common to ecological data (Cutler et al., 2007; Evans & Cushman, 2009; Evans, Murphy, Holden, & Cushman, 2011). In addition, Random Forest accommodates many predictor variables, does not assume independence of samples, and does not require a priori hypotheses regarding the direction of the response variable, thus allowing unexpected interactions to be discovered (Evans et al., 2011). Random Forest often creates highly predictive classification and regression models, but it is criticized for offering limited insight as to the mechanistic relationships between predictor and response variables (McCue, McGrath, & Wiersma, 2013; Murphy, Evans, & Storfer, 2010). However, partial plots (graphical representation of the functional relationship between predictor and response variables) and overall model significance tests have increased model interpretability (Evans & Cushman, 2009; Evans et al., 2011; Murphy et al., 2010). Indeed, Random Forest and other related machine‐learning algorithms often yield better predictions than parametric statistical models, including Generalized Linear Models (Cutler et al., 2007; McCue et al., 2013).

For each model, we compared all lynx‐used points within the burn to an equal number of random available points within the burn (Evans & Cushman, 2009). We subsampled 80% of the data using the R program Spatial Intensity Weighted Subsample (Evans, 2015) using R packages *spatialEco* (Evans, 2015), *sp* (Bivand, Pebesma, & Gomez‐Rubio, 2013; Pebesma & Bivand, 2005), and *spatstat* (Baddeley, Rubak, & Turner, 2015) leaving 20% of the data for an independent validation. We explored whether any one lynx selected habitat significantly differently from the others in a general model of habitat selection (Vanbianchi, 2015); no lynx showed significant individual variation.

We developed Random Forest models following methods outlined in Murphy et al. (2010). Specifically, we removed growing season precipitation within a large‐ and small‐scale area from the Whiteface Burn Model and growing season precipitation within a large‐scale area from the Tripod Burn Model as they were identified as multivariate redundant variables and we tested for but detected no highly collinear variables as identified by Spearman's rank test (*r* > 0.8). We identified the most parsimonious set of predictor variables for each burn model that contributed to overall model performance. For each burn model, we ran a Random Forest model using 4,000 bootstrap samples and then calculated a Model Improvement Ratio for each variable based on each variable's importance to the model. Variables with Model Improvement Ratios above increasingly high thresholds (thresholds range from 0 to 1 in 0.1 increments) were grouped. The final group of variables were chosen based on minimizing the out‐of‐bag error, the within‐class error, and the number of variables (Table S2). To insure that each fire model explained significantly more variation in the data than expected by random chance, we assessed significance by randomizing the used and available data 1,000 times to create a null distribution of model accuracy based on a random dataset. A fire model was significant if the model accuracies were significantly better than the random model accuracy distribution (Murphy et al., 2010).

## 3 Results

**Figure 3:**
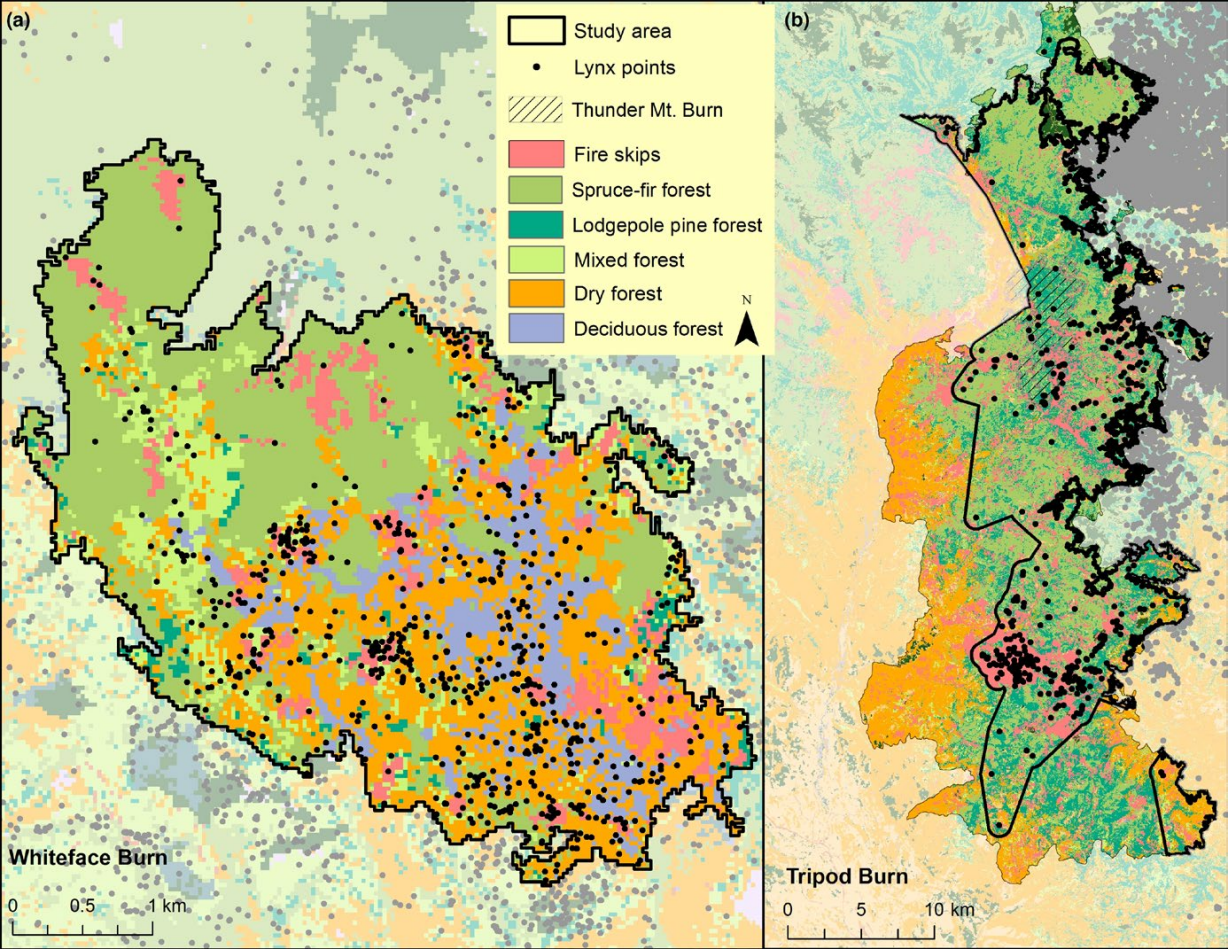
Lynx locations with two burned areas in northcentral Washington (note different scales). The Tripod Burn study area was truncated to the area used by radio‐collared lynx. (a) Lynx locations within the Whiteface Burn. (b) Lynx locations within the Tripod Burn. Habitat types and lynx locations outside the burns are shown in faded colors

**Figure 4:**
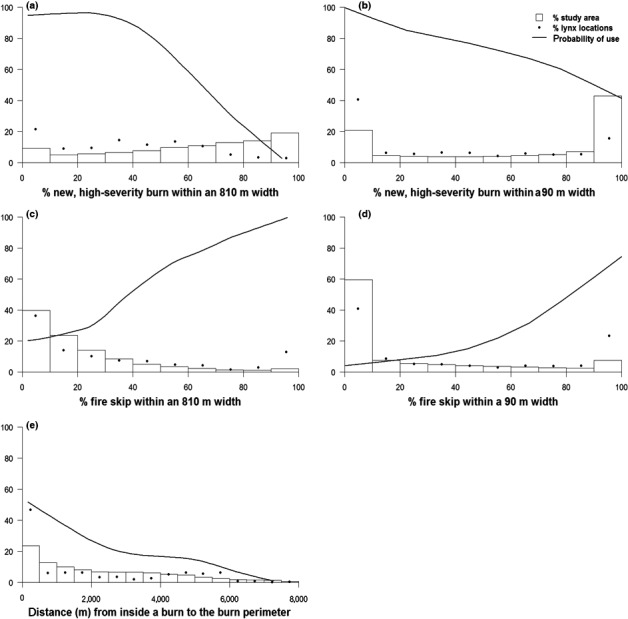
Lynx selection of habitat within the Tripod Burn. Probability of use represents the effect of a focal habitat variable on lynx habitat selection when the effect of all other habitat variables in the model is averaged. Histograms show the distribution of the focal habitat variable throughout the Tripod Burn study area. The dots represent the percentage of lynx points found within each histogram category of the focal habitat variable. Panels show lynx use of (a) new, high‐severity burn at a broad scale; (b) new, high‐severity burn at a fine scale; (c) fire skips at a broad scale; (d) fire skips at a fine scale; and (e) distance to the edge of the burn

Only 5.7% (789 of 13,972) of lynx locations near the Tripod Burn were within burned areas (Figure 3). Surprisingly, however, lynx used new burned areas regularly, entering them as early as 1 year postfire, and were able to make the best of the burn by selecting habitat characteristics that provided cover. The majority of the lynx points within the burn were not the result of multiday forays but rather were individual fixes sandwiched between locations in mature forest. Within the Tripod Burn, the top predictors of lynx locations were variables describing burn severity or distance from the edge of the burn (Figure 4, Figure S1). Within the Tripod Burn, lynx selected areas near to residual trees or fire skips, especially a large island of regenerating trees that resulted from the 1970 Forks fire (1,850 ha). In addition, 79% of the lynx locations within the Tripod Burn were <1,000 m from the fire perimeter or in or near a patch of residual trees. Variable importance scores show that lynx avoided areas of recent high‐severity burn and areas further than ~500 m from the burn perimeter. At a broad scale (0.66 km^2^), lynx selected for areas with fire skips and high canopy cover. At a fine scale (0.008 km^2^), lynx selected areas with residual patches. Climate, topography, and forest type selection patterns were of much less importance than selection explained by burn variables (Figure S1).

**Figure 5:**
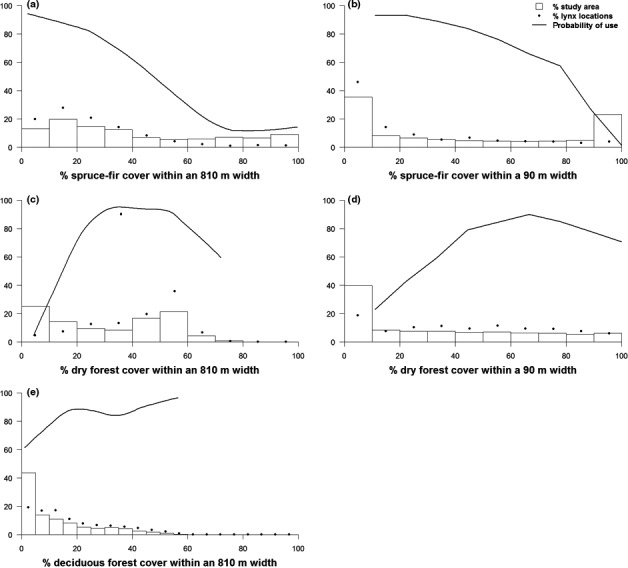
Lynx selection of forest types in the Whiteface Burn study area. Probability of use represents the effect of a focal habitat variable on lynx habitat selection when the effect of all other habitat variables in the model is averaged. Panels show lynx selection for (a) spruce‐fir forest at a broad scale; (b) spruce‐fir forest at a fine scale; (c) dry forest at a broad scale; (d) dry forest at a fine scale; and (e) deciduous forest at a broad scale

**Figure 6:**
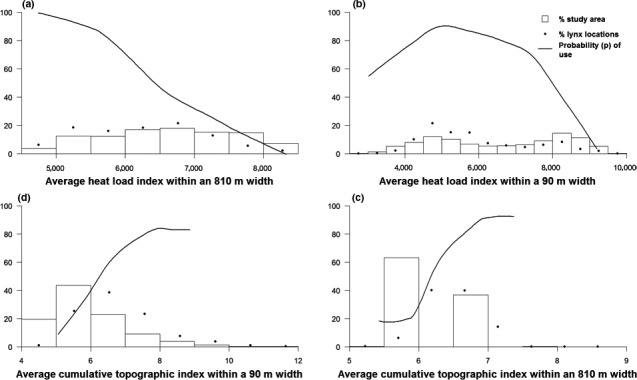
Lynx selection of climate in the Whiteface Burn study area. Probability of use represents the effect of a focal habitat variable on lynx habitat selection when the effect of all other habitat variables in the model is averaged. Panels show lynx selection for (a) average heat load at a broad scale; (b) average heat load at a fine scale; (c) average cumulative topographic index at a broad scale; and (d) average cumulative topographic index at a fine scale

In contrast, lynx selected older burns; all five lynx near the older 1994 Whiteface Burn used it, with 11% (765 points of 6,772) of their locations within the burn (Figure 3). Top predictors in the older 1994 Whiteface Burn Model described forest types and percent canopy cover, topographic setting, and microclimates, but did not include any of the variables describing burn severity (Figure 5). Distance from perimeter was not included in the final Whiteface Burn Model; lynx occurred across the entire area, and there is no evidence for lynx preferring to be nearer the unburned habitats adjacent to the burn. The lack of an edge signature in the Whiteface Burn means lynx were responding to structure within the burn, not to the perimeter. Spruce‐fir and dry forest were the most important broad‐scale predictors of lynx locations. Lynx avoided spruce‐fir cover but selected dry forest and areas with deciduous forests at a broad scale (Figure 5). Habitat quality also varied according to microclimate as cool, moister areas supported denser regeneration (Casady, van Leeuwen, & Marsh, 2010; Crotteau et al., 2013; Lillybridge et al., 1995) and provided the high‐cover habitats lynx selected regardless of forest type. Dry forest cover within a small‐scale area was also selected by lynx, although this variable's importance was less than that of dry forest at a broad scale (Figure S1). Similarly, spruce‐fir forest was also avoided within a small‐scale area and was of less importance than at a broad scale. Additional explanatory variables in the Whiteface Burn were low heat load values found on shallow, northeast‐facing slopes and moist sites as depicted by the Compound Topographic Index (Gessler et al., 1995; Moore et al., 1993), indicating lynx use of areas with more moisture (Figure 6).

## 4 Discussion

The patterns of habitat use revealed in this study can be distilled to a single overarching theme: Forest structure allows lynx to use areas of new burns and thrive in old burns. Useable structure can be residual living trees left in a recent burn or areas of dense forest regeneration in an old burn. While previous studies have shown snowshoe hares and lynx use dense understory structure in undisturbed forest, our results highlight an even more critical importance of forest structure for lynx venturing into burned areas.

### 4.1 The new Tripod Burn

Although our results confirm the overall low probability of lynx using new, high‐severity burned areas (Koehler et al., 2008; Mowat & Slough, 2003; Paragi et al., 1997), our large and detailed dataset was able to detect rarer habitat uses to reveal that lynx made the most of the Tripod Burn area immediately postfire, which contradicts previous assumptions that new burns have no value as lynx habitat. Lynx made the most of the Tripod Burn by selecting suitable fire skips as hunting habitat where islands of unburned forest remained quality hare habitat (Lewis et al., 2011), and more marginal residual cover for traveling across otherwise open‐burned areas (Vanbianchi, 2015; Vanbianchi et al., unpublished data). Lynx primarily used residual forest structure in areas <550 m from the burn perimeter, and one lynx also regularly used a large fire skip over 5 km from the burn perimeter. Use of this and other fire skips demonstrates the usefulness and importance of large patches of quality habitat contained within burns and corroborates previous observations in this region of lynx using islands of young trees that supported snowshoe hares within a 10‐year‐old burn (Lewis et al., 2011). Further demonstrating the importance of residual cover to lynx in new burns, forest cover types were not highly predictive of lynx use in the Tripod Burn: Anything that offered cover was used. The relative unimportance of forest cover type within burns contrasts with mature forests where the presence of boreal forest types is highly predictive of lynx use (Koehler et al., 2008; Vanbianchi, 2015; Vanbianchi et al., unpublished data).

### 4.2 The old Whiteface Burn

Lynx use of the Whiteface Burn centered around forest structure. However, rather than lynx depending on postfire residual structure as in the new, Tripod Burn, the 20‐year‐old Whiteface Burn, had largely regenerated enough that lynx were able to use areas of dense regeneration in addition to fire skips. Indeed, results from additional habitat models revealed that much of the Whiteface Burn provided high‐quality core lynx habitat, although habitat quality varied and not all areas supported core lynx habitat (Vanbianchi, 2015; Vanbianchi et al., unpublished data).

Lynx in the Whiteface Burn favored areas where cool and moist growing conditions supported thick understory cover. Similar to habitat selection in the Tripod Burn, the importance of dense forest cover outweighed the importance to lynx of boreal forest types in undisturbed forests: lynx in the Whiteface Burn selected *for* the normally avoided dry forest and *against* spruce‐fir forests. A field examination of the Whiteface Burn explained this interesting switch in lynx‐selected forest type. At the northern end of the Whiteface Burn, sub‐boreal climate conditions support the regeneration of spruce‐fir forests, while at the southern, lower‐elevation end of the Whiteface Burn, dry forest regeneration is common. Spruce‐fir regeneration at the northern end of the burn is short and sparse, and sub‐boreal forest regeneration in the Whiteface Burn thus provides little cover for snowshoe hares and lynx. In contrast, at the southern end of the burn and especially in draws, large amounts of willow (*Salix* spp.) and alder (*Alnus* spp.) are mixed with Douglas fir and ponderosa pine trees in the regenerating dry forests. The densely growing deciduous species provide thick understory cover for snowshoe hare and lynx, which matches findings by Mowat and Slough (2003) that lynx and snowshoe hares in the Yukon selected dense willow patches. By selecting the dry forests lynx usually avoid (Maletzke et al., 2008; Vanbianchi, 2015; Vanbianchi et al., unpublished data), lynx in the Whiteface Burn demonstrated the importance of thick understory structure over forest type for lynx habitat, confirming prior research (Mowat & Slough, 2003).

Our results clearly demonstrate that residual forest cover, especially fire skips, allow lynx to use new burns and that as burns regenerate, microclimates conducive to growing dense cover create rich lynx habitat. However, as climate change progresses and summers in the boreal region become drier and warmer, the wildfire season is predicted to become even longer and more severe (Balshi et al., 2009; Fauria & Johnson, 2007; Littell et al., 2010), with more frequent fires burning larger areas at higher severity (Hessburg et al., 2015). Not only will this ongoing regime shift cause more lynx habitat to revert to the open stand‐initiation stages (O'Hara, Latham, Hessburg, & Smith, 1996) that snowshoe hares and lynx generally avoid, but also higher severity burns may homogenize areas within a burn perimeter so that the residual trees and fire skips lynx select are less abundant (Cansler & McKenzie, 2014). Additionally, climate change may also degrade regenerating lynx habitat in burns as warmer and drier summers will likely hinder the regeneration of dense forest stands (Littell et al., 2010).

The finding that lynx are able to use areas of new burns offers hope to lynx in increasingly burned landscapes and corroborates a recent study that indicates lynx occupancy is affected by habitat loss but not by habitat fragmentation on a landscape scale (Hornseth et al., 2014). These authors suggest that in central Ontario, lynx adapted their habitat selection patterns so that fragmentation of quality lynx habitat did not affect lynx occurrence; lynx were able to adapt to local habitat conditions and use small patches of resources, thus surviving in fragmented landscapes (Hornseth et al., 2014). However, a tipping point must exist in burned landscapes past which the amount of useable fire residuals does not compensate for the amount of habitat lost to new, high‐intensity burned areas and lynx populations suffer. Discovering where this tipping point exists is an area for further exploration. Furthermore, while lynx have the ability to occupy home ranges and a broader landscape fragmented by disturbances, how different habitats and habitat configurations affect population dynamics is unknown for lynx in Washington. Indeed, a recent study in Montana found that reproductive success was highest for female lynx living in home ranges with more continuous high‐quality habitat (Kosterman, 2014). Additionally, snowshoe hares in the North Cascades are sensitive to matrix habitat types and hare densities are highest in continuous habitat or in habitat patches surrounded by matrix habitat more similar to core forest habitats (Lewis et al., 2011). Using a spectrum of habitat types may allow lynx to exist in the North Cascades, but questions remain regarding how lynx population dynamics are affected by more frequent wildfires, and a prey species that is also sensitive to more open habitats. As climate change increases the amount of recently burned areas in boreal landscapes, discovering how lynx population dynamics are affected by wildfires becomes urgent.

### 4.3 Implications for forest predators and fire management

In terms of conservation of forest carnivores, our findings offer a mixed message. First, lynx use burned landscapes more often and more rapidly postfire than previously thought, which offers some hope that lynx and potentially other forest carnivores are resilient to large disturbances (see also Fisher & Wilkinson, 2005). In contrast, if fire regimes do shift such that landscapes are more frequently burned by severe fires than in the past, leading to high proportions of landscapes in early‐seral conditions, there may be inadequate mature forest, postfire residuals, or regrowth to sustain predators in these heavily burned landscapes. Although we have focused on the highly vulnerable Washington lynx population, which is thought to number <100 individuals (Lewis, 2016), we note that regime shifts in fires are also likely affecting lynx and other predators within Montana, Wyoming, and Colorado. We suspect other forest predators likewise depend on postfire heterogeneity in order to make use of burned landscapes, although research on predators and fire is quite limited (Fisher & Wilkinson, 2005).

Current forest and fire management involves many practices that may be damaging the ability of predators to use postfire landscapes. Areas disturbed by wildfires are not uniform. Instead, wildfires create a diversity of habitat conditions that depend upon burn severity and microclimates that influence forest regeneration rates and patterns. In turn, lynx respond to burned areas with habitat selection patterns that are more nuanced than previously described patterns for lynx in harvested areas (Simons‐Legaard, Harrison, Krohn, & Vashon, 2013). The heterogeneous habitats created by wildfires are in contrast to disturbed habitats created by timber harvest which, even when designed to emulate a fire disturbance, create more uniform patterns of disturbance with less edge area and fewer standing live trees left after harvest (McRae, Duchesne, Freedman, Lynham, & Woodley, 2001). Regeneration patterns between burned areas and harvested areas also differ as residual trees and coarse woody debris left postfire can seed and protect young seedlings (Brassard & Chen, 2006). Furthermore, cycles of harvest are often shorter than burn cycles and occur over smaller areas (McRae et al., 2001). Lynx and snowshoe hares avoid harvest conditions that eliminate understory cover, such as thins and new clear‐cuts, but are benefitted by old clear‐cuts that promote thick forest regeneration (Simons‐Legaard et al., 2013; Squires et al., 2010). In contrast to this relatively simple and more predictable response, the heterogeneous habitat created by burns provides lynx with more varied habitats to suit their survival needs than areas disturbed by timber harvest, especially in new burns where fire skips and low‐severity burns create cover for lynx.

Treatments such as burn‐out operations of dead fuels and fire skips to avoid new spot fires during a fire or postfire salvage logging reduce habitat quality, with road building, soil compaction, and removal of residual living and dead tree biomass (Lindenmayer, Burton, & Franklin, 2008; Peterson et al., 2009). Salvage logging slows and alters tree regeneration in the immediate years postfire, as well as removing existing vegetation that might act as habitat or cover for some species (Boucher, Gauthier, Noel, Greene, & Bergeron, 2014; Donato et al., 2006). Because fire skips are important habitat constituents for lynx after fires, lynx conservation would be aided by preventing burn‐outs, reducing postfire salvage logging, and ensuring that trees that survived the fire are protected. For example, Colorado may salvage log thousands of square kilometers of beetle‐killed forest in an effort to reduce fuels and alter fire sizes and severity (USDA 2015), but the salvage itself may damage lynx habitat if residual trees are eliminated.

We therefore agree with recent advances in fire and landscape ecology: Forest management needs to change before fire, during firefighting, and after fires, if we are to sustain forest mosaics that contain appropriate amounts and configurations of different stand types that predators and their prey can use (Hessburg et al., 2015; Perry et al., 2011). Prefire, management tools include fuels reductions, harvest, and prescribed fires, all as ways to affect where large wild fires might burn. Managers could prescribe burns and craft timber harvest units that would act as natural fire breaks to decrease the spread and intensity of increasingly severe fires, thus preserving heterogeneous burn patterns that provide cover for predators. Similarly, forest management that promotes cooler, less severe fires may lead to a faster postfire return of suitable habitat conditions for hares and lynx. During fires, firefighting decisions about where to deploy defenses will likewise affect the size, shape, and severity of each fire. Postfire, salvage logging is an additive disturbance that may be a direct threat to carnivore conservation if it removes residual structures the animals could otherwise use to access recent burns and hinders regeneration. We suggest that landscape‐scale planning that affects the distribution of fire sizes and severities will be essential to ensure predators remain on these increasingly fire‐disturbed landscapes.

## Author Contributions

CMV assembled the GIS layers, analyzed the GIS and telemetry data, and wrote the initial draft. MAM advised on the use of R and Random Forest models and provided editorial comments on the manuscript. KEH initiated the idea; secured data, funding, and agency collaborations; and helped write the paper. All authors were essential for this paper and approved its final form.

## Data Accessibility

Data are archived at the University of British Columbia and the Washington Department of Fish and Wildlife, as per government requirements.

## Conflict of Interest

None declared.
