# Supplementary material for: Canada lynx use of burned areas: Conservation implications of changing fire regimes
Source: Ecol Evol. 2017 Mar 12;7(7):2382–94. doi: 10.1002/ece3.2824 (PMC5383493; doi:10.1002/ece3.2824)
Supplement: Supplementary file 1 [file ECE3-7-2382-s001.docx]

**Supporting Information**

Online, we show details about the models we constructed. Figure S1 shows importance plots for the variables retained in the models. Table S1 offers details for each variable and the scales used for each variable. Table S2 summarizes model fit statistics.


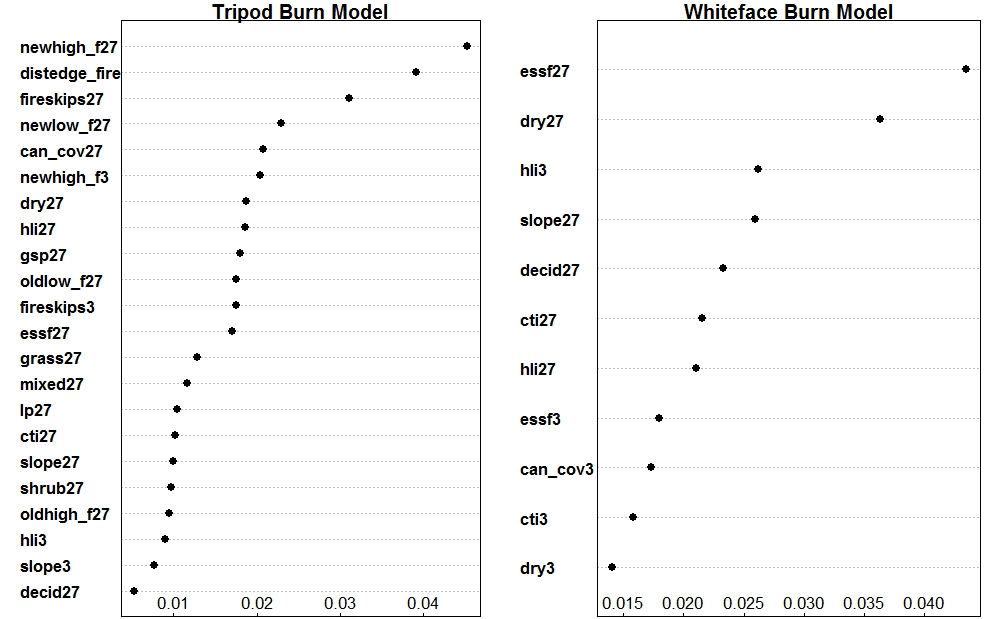


**Figure S1.** Importance plots for the Tripod and Whiteface Burn Models. Plots show the ranking of each habitat variable retained in the final Random Forest models. Values of out-of-bag observations were randomly permuted, run down each tree, and predicted as used or available. The misclassification rate of the modified out-of-bag observations was subtracted from the misclassification rate of the unmodified out-of-bag observations and divided by the standard error. Numbers after the variable name identify it as being portrayed at a broad scale (27x27 pixel area) or fine scale (3x3 pixel area). Variables are explained in Table S1. Note different x-axes.

**Table S1.** Habitat variables used in the Random Forest models of lynx habitat use in burned areas.

| Variable Class | Variable | Layer Name | Measurement Type |
| --- | --- | --- | --- |
| Land Cover^a^ | Lodgepole pine | lp | Number of like pixels within a 3x3 or 27x27 window |
|  | Spruce-fir | essf |  |
|  | Mixed forest | mixed |  |
|  | Dry forest | dry |  |
|  | Deciduous forest | decid |  |
|  | Ice or rock | rock_ice |  |
|  | Grassland | grass |  |
|  | Shrub land | shrub |  |
|  |  |  |  |
| Disturbance^b^ | Old, high-severity fire | oldhigh_f | Number of like pixels within a 3x3 or 27x27 window |
|  | Old, low-severity fire | oldlow_f |  |
|  | New, high-severity fire | newhigh_f |  |
|  | New, low-severity fire | Newlow_f |  |
|  | Fire skips | fireskips |  |
|  |  |  |  |
| Patch Metrics^b^ | Distance to edge of fire | distedge_fire | Meters |
|  |  |  |  |
| Topography^c^ | Slope | slope | Average slope at 3x3 or 27x27 window |
|  | Distance to nearest draw | dist_draw | Meters |
|  |  |  |  |
| Climate | Compound Topographic Index^c^ | cti | Lower numbers indicate wetter areas, averaged across a 3x3 or 27x27 window |
|  | Heat Load Index^c^ | hli | Index, lower numbers indicate warmer areas. Index averaged across a 3x3 or 27x27 window |
|  | Growing season precipitation^d^ | gsp | Average total precipitation (mm) between April and September. Index averaged across a 3x3 or 27x27 window |
|  |  |  |  |
| Forest Structure^a^ | Canopy cover | can_cov | Average percent canopy cover in a 3x3 or 27x27 window |
|  |  |  |  |

Original data source used to develop each layer:

^a^ GNN Species Size, developed by Landscape Ecology, Modeling, Mapping and Analysis (LEMMA). Land cover layers optimized using US Geological Survey, Gap Analysis Program data developed by the Northwest Gap Analysis Project.

^b^ LandTrender disturbance intensity data developed by the Laboratory for Applications of Remote Sensing in Ecology, Oregon State University, and the Pacific Northwest Research Station. Fire perimeters defined using the data layer S_R06.FireHistoryPL developed by Data Resource Management/Fire and Aviation, Pacific Northwest Region, US Forest Service, baselayer.BL_VECTOR.US_HIST_FIRE_PERIMTRS_DD83_NEW developed by Geospatial Multi-Agency Coordination Group, and Wa_historical_fires_1973_2012 developed by the Washington Department of Natural Resources, USDA Forest Service, and the Bureau of Land Management. FragStats 4.1 (McGarigal, Cushman, & Ene, 2012) used to create the distedge_fire layer.

^c^ US Geological Survey 30 m National Elevation Dataset. Slope and dist_draw developed using the ArcGIS 10.1 Slope and Flow Accumulation tools respectively (ESRI 2012). HLI and CTI were developed using Geomorphometry and Gradient Metrics tools (Evans 2011).

^d^ Growing Season Precipitation, April to September. Developed by the Moscow Forestry Sciences Laboratory.

**References**

McGarigal, K., Cushman, S.A., & Ene, E. (2012) FRAGSTATS v4: Spatial Pattern Analysis Program for Categorical and Continuous Maps. <http://www.umass.edu/landeco/research/fragstats/fragstats.html>.

Evans, J. (2011) ArcGIS - Geomorphometry and Gradient Metrics Toolbox. Retrieved December 1, 2013, from <http://evansmurphy.wix.com/evansspatial#!arcgis-gradient-metrics-toolbox/crro>.

ESRI (2012) ArcGIS 10.1. Redlands, CA.

**Table S2.** Model fit and validation statistics for the Tripod and Whiteface Burn Models. Out-of-bag error rates (%) show the mean misclassification rate of trees when predicting the out-of-bag data across Random Forest bootstrap. Accuracy (%) indicates the overall performance of the model when predicting the withheld, validation dataset. Sensitivity and specificity show the proportion of used locations correctly predicted and the proportion of available locations correctly predicted. Area under the curve of a Receiver Operator Characteristic (AUC) scores are a measure of how evenly the model predicts sensitivity and specificity. The Kappa (k) statistic is a measure of how much better the model predicted used and available points than expected by random chance and P values indicate significance of each model.

|  | Model  Fit | Model Validation Statistics | | | | | |
| --- | --- | --- | --- | --- | --- | --- | --- |
| Model | Out-of-bag error (%) | Accuracy (%) | Sensitivity | Specificity | k | AUC | P value |
| Tripod Burn | 21.67 | 75.60 | 0.7457 | 0.7673 | 0.51 | 0.7560 | < 0.001 |
| Whiteface Burn | 30.96 | 70.92 | 0.7759 | 0.6684 | 0.42 | 0.7091 | < 0.001 |
|  |  |  |  |  |  |  |  |
